# Increased similarity of neural responses to experienced and empathic distress in costly altruism

**DOI:** 10.1038/s41598-019-47196-3

**Published:** 2019-07-24

**Authors:** Katherine O’Connell, Kristin M. Brethel-Haurwitz, Shawn A. Rhoads, Elise M. Cardinale, Kruti M. Vekaria, Emily L. Robertson, Brian Walitt, John W. VanMeter, Abigail A. Marsh

**Affiliations:** 1 Interdisciplinary Program in Neuroscience, Georgetown University, Washington, DC 20057 USA; 2 Department of Psychology, University of Pennsylvania, Philadelphia, PA 19104 USA; 3 Department of Psychology, Georgetown University, Washington, DC 20057 USA; 4 National Institute of Nursing Research, National Institutes of Health, Bethesda, MD 20892 USA; 5 Department of Neurology, Georgetown University Medical Center, Washington, DC 20057 USA

**Keywords:** Empathy, Human behaviour

## Abstract

Empathy—affective resonance with others’ sensory or emotional experiences—is hypothesized to be an important precursor to altruism. However, it is not known whether real-world altruists’ heightened empathy reflects true self-other mapping of multi-voxel neural response patterns. We investigated this relationship in adults who had engaged in extraordinarily costly real-world altruism: donating a kidney to a stranger. Altruists and controls completed fMRI testing while anticipating and experiencing pain, and watching as a stranger anticipated and experienced pain. Machine learning classifiers tested for shared representation between experienced and observed distress. Altruists exhibited more similar representations of experienced and observed fearful anticipation spontaneously and following an empathy prompt in anterior insula and anterior/middle cingulate cortex, respectively, suggesting heightened empathic proclivities and abilities for fear. During pain epochs, altruists were distinguished by spontaneous empathic responses in anterior insula, anterior/mid-cingulate cortex and supplementary motor area, but showed no difference from controls after the empathy prompt. These findings (1) link shared multi-voxel representations of the distress of self and others to real-world costly altruism, (2) reinforce distinctions between empathy for sensory states like pain and anticipatory affective states like fear, and (3) highlight the importance of differentiating between the proclivity and ability to empathize.

## Introduction

Fewer than 350 Americans annually undergo surgery to donate a kidney to a stranger (Organ Procurement and Transplantation Network Data as of May, 2019). These donations represent extraordinary examples of *altruism*: a voluntary, costly behaviour aimed at benefiting the well-being of another individual^1^. Traditional models of social behaviour struggle to accommodate costly altruism for strangers^2^, but recent research suggests that individuals who engage in such behaviour exhibit enhanced responsiveness to others’ distress^3–5^. These findings are broadly consistent with the *empathy-altruism hypothesis*, whereby affective resonance with other individuals’ distress can give rise to empathic concern and prosocial motivation^6^.

Neuroimaging investigations of empathy have typically focused on responses to sensory pain. Univariate analyses consistently show overlapping responses in regions that encode affective and motivational features of pain, particularly anterior insula (AI) and dorsal anterior cingulate/anterior midcingulate cortex (dACC/aMCC), during experienced and observed painful events^7–13^. Analgesic manipulations reduce both experienced and empathic pain responses in AI and dACC/aMCC^14,15^ and multi-voxel pattern analyses (MVPA) have found similar patterns of responses in these regions during experienced and observed pain^16,17^, ^but see^ ^18^, suggesting that empathic pain involves neural processes that are similar to the first-hand experience of pain. Activity within AI also scales parametrically with self-reported empathy^19,20^ and, consistent with the *empathy-altruism hypothesis*, empathic AI responses correspond to motivation to reduce a stranger’s pain in the laboratory^20^.

Previous work in our laboratory has found greater overlap of univariate BOLD responses during experienced and observed pain in bilateral AI and dACC/aMCC in extraordinary altruists relative to controls^5^. Altruists also exhibit greater self-other overlap in AI during the fearful anticipation of pain, consistent with prior work linking altruism to increased sensitivity to others’ fear^3,21,22^. However, it remains unknown whether these overlapping responses reflect more similar multi-voxel patterns, which would suggest true abstraction across experienced and observed distress and more directly index empathic affective resonance. Our first goal was therefore to use multivariate cross-classification (MVCC)—a machine learning approach in which a classifier is trained to discriminate between neural responses in one context then tested for its ability to discriminate between responses in a distinct context^23^—to examine the role of affective resonance in altruism. We used this approach to assess the similarity of altruists’ experienced and observed (empathic) multi-voxel response patterns to both pain and fearful anticipation. Cross-classification between experienced and observed responses would suggest shared self-other representation, indicative of affective resonance. We used MVCC to compare responses to experienced and observed pain and fearful anticipation in real-world altruists and controls both at baseline and following a verbal empathy-induction prompt. Because weak empathic responding can in some cases be mitigated by instructional prompts^24,25^, it has been suggested that real-world altruism may merely reflect an increased propensity to spontaneously empathize. Therefore, our second goal was to use instructional prompts to test whether altruists have an enhanced ability to empathize with others’ distress, which would be indicated by enhanced self-other mapping in altruists even following prompts instructing all participants to empathize.

**Figure 1:**
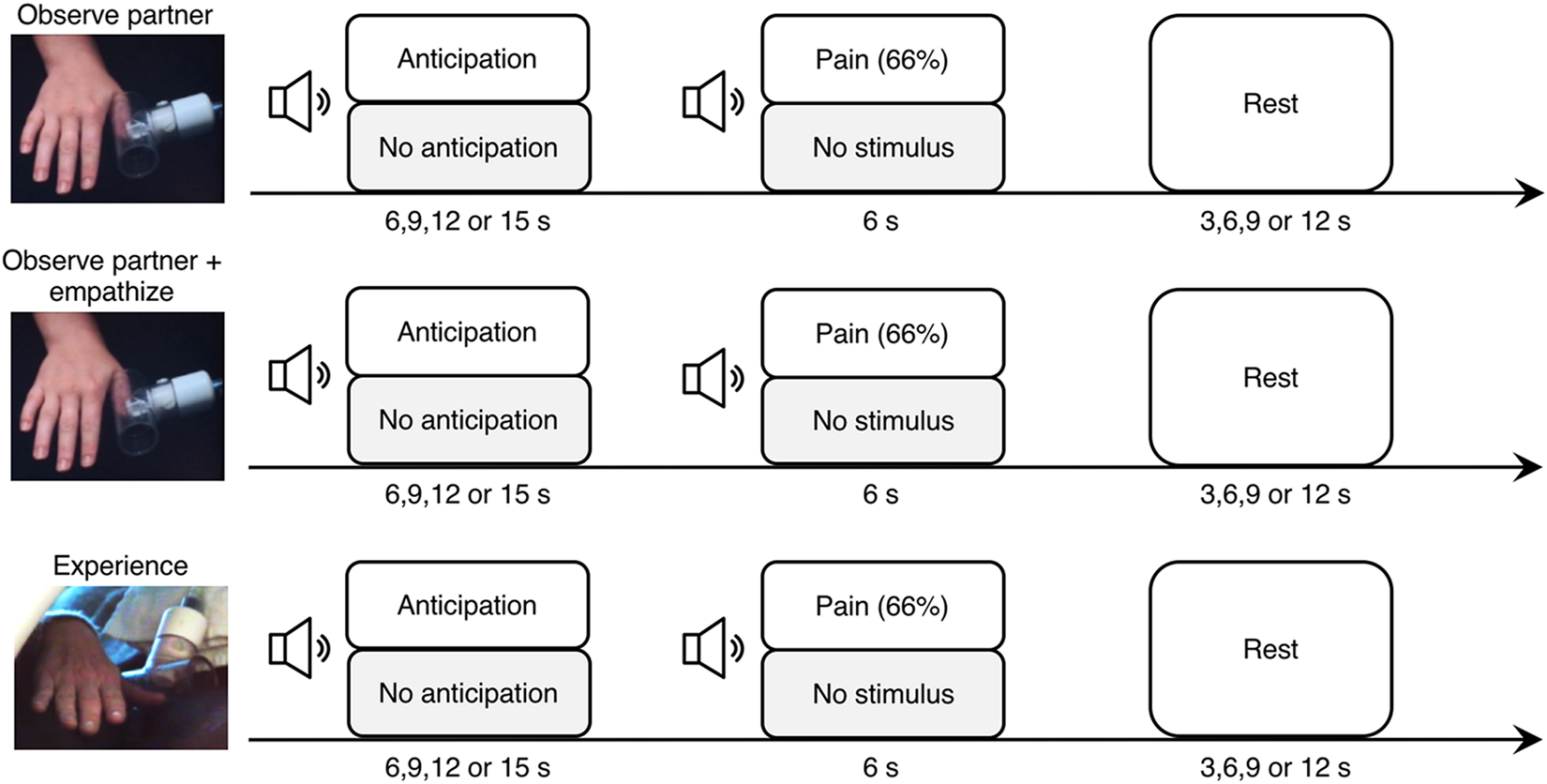
Experimental design. Trials were introduced by an audio cue, which signaled either an aversive or non-aversive trial type. A second audio cue signaled the onset of the painful stimulus in the aversive trial type, and had no meaning during the non-aversive trial type. In the first run of the experiment, subjects viewed their study partner experiencing the task. The second run was identical to the first, except for the addition of a verbal empathy prompt prior to the task. During the third run, subjects experienced the stimulus first-hand.

Fifty-seven male and female subjects, including 29 altruistic kidney donors and 28 demographically matched controls, underwent a pressure-pain task during functional neuroimaging (Fig. 1). During the first run of the task, all subjects watched as a stranger they had been briefly introduced to (but had not interacted with) anticipated and experienced pressure-pain stimulation to the thumbnail of their right hand. In the second run of the task, subjects observed the stranger anticipating and experiencing pain after being verbally prompted to empathize with them with the instructions: “*Please watch your partner during the following session of the task closely. As you watch and listen, please imagine how your partner is feeling during the task. Really try to understand her thoughts and emotions during each trial of the task*”^24^. Finally, in the third run of the task, subjects anticipated and experienced the pressure-pain stimulation to their right thumbnail. Non-aversive control trials were included throughout each of the three runs.

An MVCC approach was used to test for shared representation between neural responses to self and others’ distress in each subject. A linear support vector machine (SVM) classifier was trained on neural response patterns during one context and tested on response patterns during a distinct context (i.e. trained on *experienced* pain versus no-pain then tested on *observed* pain versus no-pain). We performed each cross-classification analysis twice, bidirectionally training and testing each context and averaging the result, consistent with prior approaches^17,26,27^. In all cross-classification analyses, training data and testing data were fully independent (from different runs) and performance was assessed using area under the receiver operating characteristic curve (AUC).

## Results

### Self-other cross-classification of fearful anticipation

**Figure 2:**
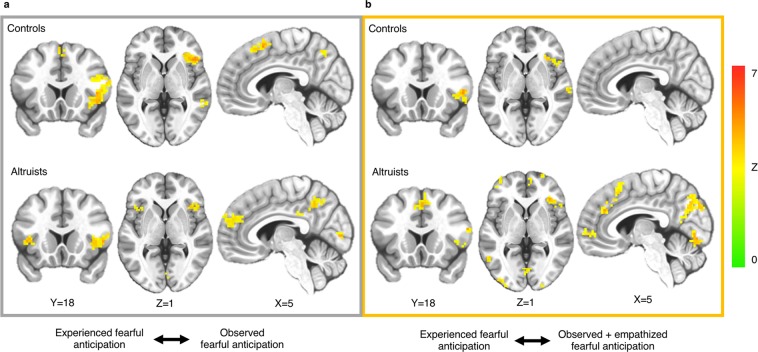
Cross-classification of experienced and observed fearful anticipation. (**a**) Both groups revealed above chance cross-classification between experienced and observed fearful anticipation in regions including right AI/IFG, TPJ and precuneus. (**b**) Cross-classification between experienced and observed fearful anticipation after an empathy prompt. p < 0.05_corr_; underlying voxel height threshold p < 0.001. Right = right.

**Figure 3:**
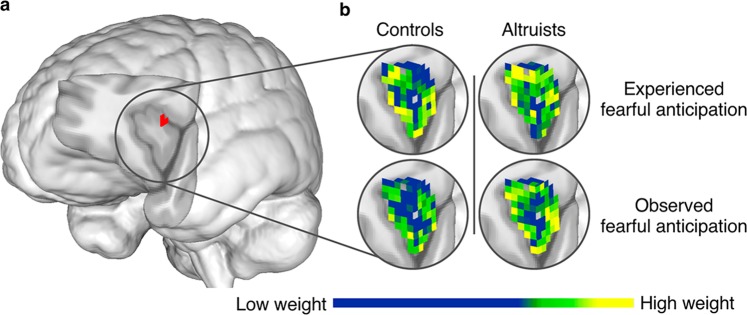
Altruists’ increased cross-classification between experienced and observed fearful anticipation in AI. (**a**) Altruists had increased cross-classification between experienced and observed fearful anticipation of pain in left AI; p < 0.05_SVC_, underlying voxel height threshold p < 0.001. (**b**) SVM pattern weights indicate how distributed voxel patterns carry information discriminating fearful anticipation events from their non-aversive control; scale represents 0–0.53.

Altruists exhibited self-other cross-classification between experienced and observed fearful anticipation of pain in multiple regions (Fig. 2a, Supplementary Table 1). These regions included bilateral AI/inferior frontal gyrus (IFG), dACC/aMCC, left temporoparietal junction (TPJ; BA39), dorsomedial prefrontal cortex (BA9), posterior cingulate cortex (PCC), precuneus and right superior parietal lobule (BA7). In controls, self-other cross-classification was observed in regions that included right AI/IFG, right TPJ (BA22/39/40), precuneus, supplementary motor area (SMA; BA6) and right dorsolateral prefrontal cortex (BA9). A group comparison confirmed that altruists exhibited more robust shared representations of fearful anticipation in left AI than did controls (p < 0.05_SVC_; Fig. 3a), indicating that this region more similarly represents experienced and observed fear in altruists. This group difference persisted even after including subjects’ final PSI pain stimulus level as a covariate of no interest. No region emerged in which controls exhibited significantly greater cross-classification.

### Self-other cross-classification of pain

**Figure 4:**
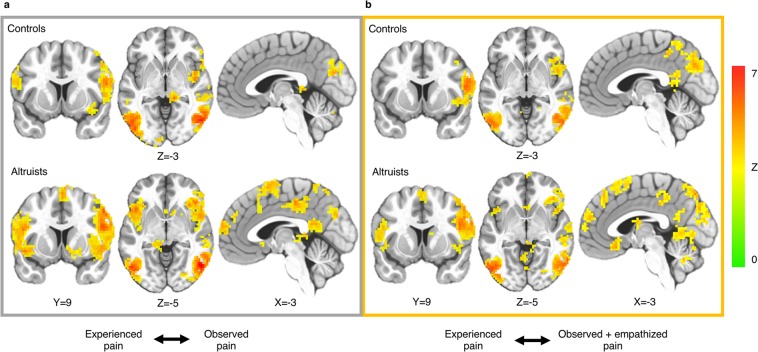
Cross-classification of experienced and observed pain. (**a**) Both groups revealed above chance cross-classification between experienced and observed pain in regions including right AI/IFG, TPJ, somatosensory cortex, motor cortex, superior parietal lobule, occipitotemporal cortex and cerebellum. (**b**) Cross-classification between experienced and observed pain after an empathy prompt. p < 0.05_corr_; underlying voxel height threshold p < 0.001. Right = right.

Both altruists and controls revealed significant self-other cross-classification between experienced and observed pain in regions including right AI/IFG, bilateral TPJ (BA22/39/40), bilateral somatosensory cortex, bilateral motor cortex, bilateral superior parietal lobule, bilateral occipitotemporal cortex and cerebellum (Fig. 4a, Supplementary Table 2). Altruists additionally showed self-other cross-classification in left AI/IFG, dorsomedial prefrontal cortex (BA9/10), SMA (BA6), PCC and nucleus accumbens.

**Figure 5:**
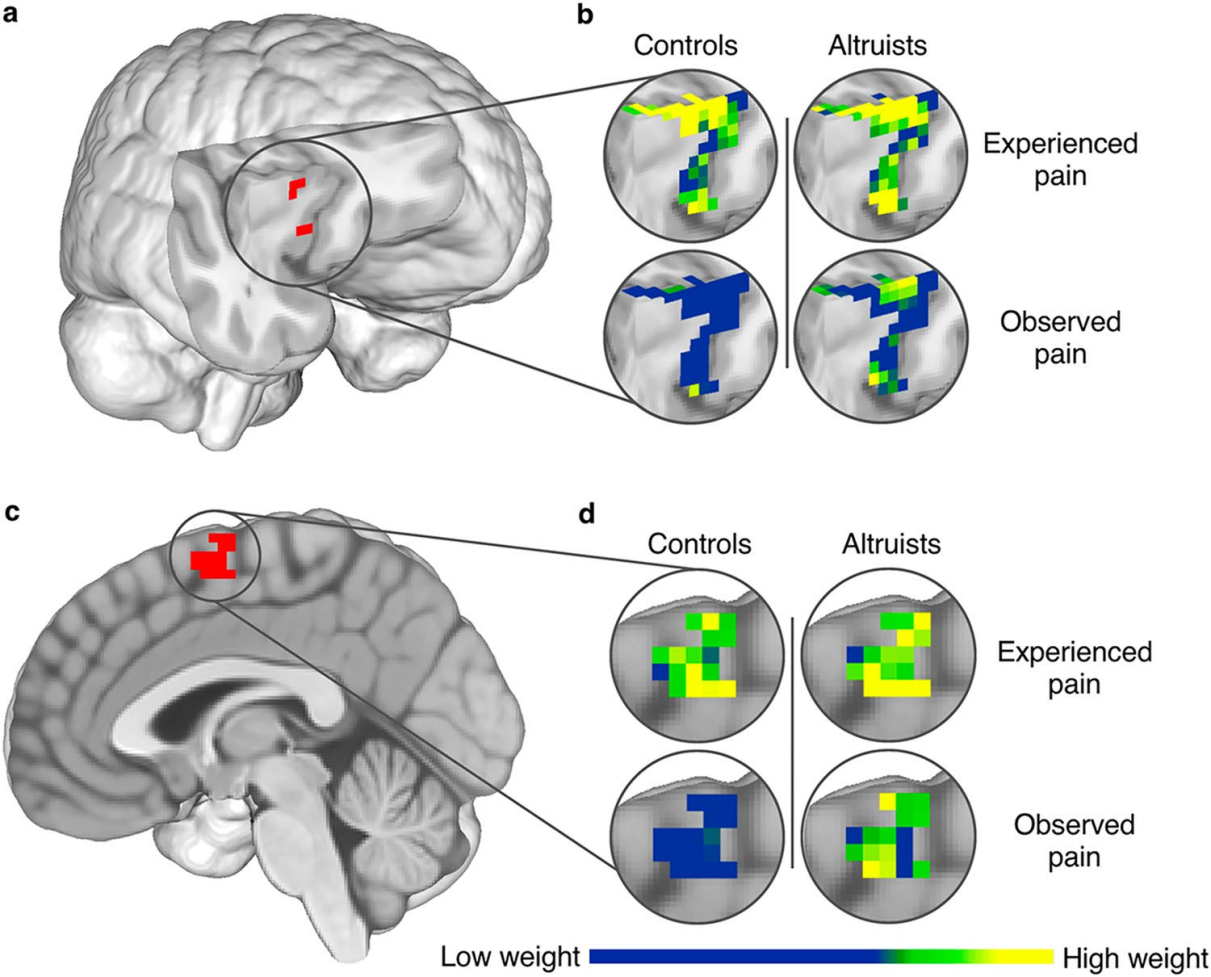
Altruists’ increased cross-classification between experienced and observed pain in AI and SMA. (**a**) Altruists had increased cross-classification between experienced and observed pain in right AI; p < 0.05_SVC_, underlying voxel height threshold p < 0.001. (**b**) SVM pattern weights discriminating pain events from their non-aversive control; scale represents 0–0.86. (**c**) Altruists also had increased cross-classification between experienced and observed pain in SMA; p < 0.05_corr_, underlying voxel height threshold p < 0.001. (**d**) SVM pattern weights discriminating pain events from their non-aversive control; scale represents 0–0.80.

More robust shared representation between experienced and observed pain was observed in altruists relative to controls in right AI (p < 0.05_SVC_; Fig. 5a), dACC/aMCC (p < 0.05_SVC_), and SMA (p < 0.05_corr_; Fig. 5c). Group differences in these regions persisted when including subjects’ final PSI pain stimulus level as a covariate of no interest. No region emerged in which controls exhibited significantly greater cross-classification.

### Fearful anticipation and pain self-other cross-classifications following the empathy prompt

**Figure 6:**
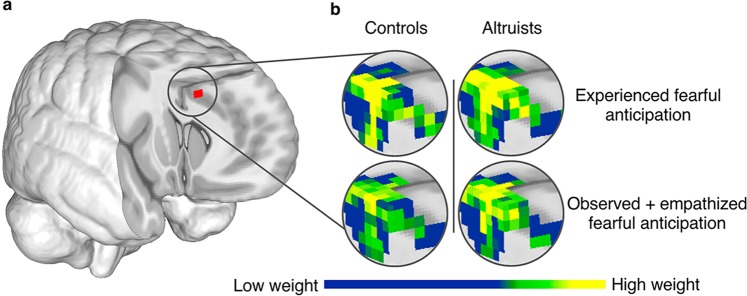
Altruists’ increased cross-classification between experienced and observed fearful anticipation after an empathy prompt in dACC/aMCC. (**a**) Altruists had increased cross-classification between experienced and observed fearful anticipation after the empathy prompt in dACC/aMCC; p < 0.05_SVC_, underlying voxel height threshold p < 0.001. (**b**) SVM pattern weights discriminating fearful anticipation events from their non-aversive control; scale represents 0–0.46.

We next assessed cross-classification between experienced and observed fearful anticipation following the prompt to empathize (Fig. 2b, Supplementary Table 3). This condition was included to identify whether altruists exhibit a greater ability to empathize than controls, which would indicate they are not merely distinguished by greater empathic proclivities. Altruists exhibited cross-classification between experienced and observed fearful anticipation in regions that were similar to those observed prior to the prompt and included right AI/IFG, dACC/aMCC, bilateral TPJ (BA39/40), dorsomedial prefrontal cortex (BA10) and precuneus, whereas cross-classification in controls following the empathy prompt emerged only in right AI/IFG, right middle temporal gyrus (BA21), and right motor cortex. A group difference contrast found that altruists exhibited greater cross-classification between experienced and observed fearful anticipation after the empathy prompt in dACC/aMCC (p < 0.05_SVC_; Fig. 6) and visual cortex (p < 0.05_corr_), both of which persisted after inclusion of subjects’ final PSI level as a covariate of no interest. No region emerged in which controls exhibited significantly greater cross-classification.

During pain events, both groups continued to show significant cross-classification between experienced and observed pain after the empathy prompt in right AI/IFG, bilateral TPJ (BA22/39/40), bilateral somatosensory cortex, bilateral superior parietal lobule, bilateral occipitotemporal cortex and cerebellum (Fig. 4b, Supplementary Table 4). Altruists additionally showed cross-classification in left AI/IFG, dorsomedial prefrontal cortex (BA8/9/10), SMA (BA6), subgenual cingulate cortex (BA25), and bilateral caudate. Unlike the fearful anticipation trials, however, no significant group difference in cross-classification of pain was observed following the empathy prompt.

### Subjective ratings of pain and affect

Subjective ratings of painful pressure confirmed that the stimulus was equally aversive for altruists and controls (Altruists: M = 4.48, SD = 1.23; Controls: M = 4.30, SD = 1.30; t(50) = 0.52, p = 0.603, *d* = 0.15, CI = −0.40 to 0.69). Groups also reported similar fear/anxiety during the fearful anticipation periods (Altruists: M = 3.36, SD = 1.52; Controls: M = 3.65, SD = 1.16; t(49) = 0.78, p = 0.442, *d* = 0.22, CI = −0.33 to 0.77) and similar overall unpleasantness of the aversive trials (Altruists: M = 3.60, SD = 1.38; Controls: M = 3.85, SD = 1.38; t(49) = 0.64, p = 0.527, *d* = 0.18, CI = −0.37 to 0.73). Equalizing subjective pain perception rating during stimulus titration prior to beginning the scan required calibrating altruists to a higher PSI level than controls (Altruists: M = 40.00, SD = 15.48; Controls: M = 26.48, SD = 8.30; t(50) = 3.97, p < 0.001, *d* = 1.10, CI = 0.51 to 1.68), resulting in PSI level being tested as a covariate in group comparison analyses.

No group differences were observed in altruists’ and controls’ explicit ratings of their partner’s experience. Subjective ratings of the partner’s pain were similar across groups (Observed; Altruists: M = 4.08, SD = 1.08; Controls: M = 3.93, SD = 0.83; t(50) = 0.58, p = 0.564, *d* = 0.16, CI = −0.38 to 0.71, Observed + Empathy Prompt; Altruists: M = 4.32, SD = 1.31; Controls: M = 4.30, SD = 1.17; t(50) = 0.07, p = 0.945, *d* = 0.02, CI = −0.53 to 0.56). A 2 (Group) x 2 (Prompt, No Prompt) ANOVA revealed a significant main effect of the empathy prompt on ratings of the partner’s pain (F(1,50) = 8.38, p = 0.006, η_p_^2^ = 0.144), but no interaction with group (F(1,50) = 0.38, p = 0.539, η_p_^2^ = 0.008) nor a main effect of group (F(1,50) = 0.10, p = 0.760, η_p_^2^ = 0.002). Subjective ratings of the partner’s overall fear/anxiety during the anticipation periods were similar across groups (Altruists: M = 3.48, SD = 1.39; Controls: M = 3.92, SD = 1.16; t(49) = 1.24, p = 0.222, *d* = 0.35, CI = −0.21 to 0.90) as were ratings of the overall unpleasantness of the partner’s experience during aversive trials (Altruists: M = 3.84, SD = 1.28; Controls: M = 3.81, SD = 1.58; t(49) = 0.08, p = 0.936, *d* = 0.02, CI = −0.53 to 0.57). A post-hoc analysis investigating the relationship between subjective pain ratings and neural responses to experienced and observed pain is reported in Supplementary Materials (Supplementary Table 5, Supplementary Fig. 1).

## Discussion

The present study used multivariate cross-classification to reveal greater similarity between real-world altruists’ neural representations of their own and others’ fearful anticipation and pain, indicating that individuals who engage in acts of costly altruism experience greater affective resonance with strangers’ distress. These patterns were observed in regions implicated in the affective and motivational features of fear and pain, including AI and dACC/aMCC^8,28–31^. It is notable that altruists showed enhanced similarity of experienced and observed fearful anticipation in left AI at baseline and—even following an empathy prompt—in dACC/aMCC. These results suggest that altruists are distinguished not only by their increased propensity to spontaneously empathize with others’ fear, but also an enhanced ability to do so. During the pain epochs, altruists showed enhanced similarity between response patterns to experienced and observed pain in right AI, dACC/aMCC and SMA, suggesting altruists have an increased propensity to spontaneously empathize with others’ pain. No group differences in empathic pain responding were observed following the empathy prompt however, indicating that altruists and controls were similarly able to empathize with others’ pain.

Whereas previous work on the neural basis of empathy has focused on identifying shared response patterns to experienced and observed pain, we additionally measured responses during the anticipation of pain, a condition during which participants reported moderate levels of fear and anxiety. Altruists showed increased cross-classification between experienced and observed fearful anticipation in AI, consistent with prior work linking altruism to increased empathy for fear, with highly altruistic individuals reliably showing greater neural and behavioural responsiveness to others’ fearful facial expressions^3,21,22^. Activity in AI is implicated in the prediction and detection of salient stimuli through the integration of bottom-up sensory and top-down processes^32–36^, and in orchestrating appropriate behavioural responses^37–39^. As such, AI is implicated in both fear and pain states, as well as emotional awareness more generally^40^. The present finding that altruists exhibited greater self-other cross-classification in AI during both fearful anticipation and pain conditions suggests increased similarity in the value they place on strangers’ distress relative to their own, regardless of the specific emotional context. As predicted by the *empathy-altruism hypothesis* and its contemporary interpretations^41,42^, this increased self-other mapping of distress plays a likely role in altruists’ motivation to alleviate the aversive experiences of others.

Relevant to this, it is important to note here the various ways in which empathy can be defined and how various forms of empathy are hypothesized to relate to altruism. The *empathy-altruism hypothesis* was studied most prominently by Batson and colleagues^24,43–45^, in whose work empathy was defined in terms of what is now more often called sympathy or concern rather than as affective resonance. However, the prompts he used to increase this state—from which we adapted the prompt used in our study—were explicitly aimed at increasing affective resonance, requesting that participants really try to imagine and understand another’s feelings and thoughts. Thus, although affective resonance (resonating with or understanding another’s emotions) and empathic concern (caring about their distress) are widely agreed to be distinct processes, the two are closely related, with affective resonance being one effective method for increasing empathic concern^46–48^.

In the second run of our task, verbal prompts instructed participants to imagine their partners’ feelings while the partners anticipated and experienced pain. This manipulation enabled us to explore whether altruists have a heightened ability to empathize relative to controls. The prompt resulted in increased reported perceptions of the study partner’s pain, consistent with prior work^24,44,49^. Similar prompts have been demonstrated to increase empathic responses in left AI among psychopathic offenders with low baseline empathic responding, such that these offenders were nearly indistinguishable from controls^25^. Consistent with these findings, no group differences in cross-classification between experienced and observed pain emerged after the empathy prompt. Group differences in response to fearful anticipation after the prompt did persist however, suggesting that altruism is associated with a heightened ability to empathize with others’ fear even when all participants have instructed to empathize. This finding suggests further research is needed to investigate the malleability of empathic responses to distinct emotional states and whether this property could be leveraged to increase prosocial behaviour.

Prior univariate analyses of this dataset revealed greater BOLD response overlap between experienced and observed fearful anticipation (left AI) and pain (left AI, dACC/aMCC) in altruists^5^. Our MVCC analyses extend these results and specify that altruists’ increased BOLD response overlap corresponds to greater affective resonance at the level of multi-voxel response patterns. This finding provides substantive evidence that real-world altruists represent others’ pain and distress more similarly to their own pain and distress. The present analyses also revealed increased similarity between experienced and observed pain response patterns in altruists’ SMA. Meta-analyses of empathy have indicated SMA’s involvement during the observation of emotion^7,11,50^; this region may represent the motor and behavioural drive component of empathic responding^51^ much as it organizes behavioural responses to personally experienced pain. For example, Han and colleagues found that empathy for pain facilitates motor action (the force and velocity of a button press), and that empathic responses in SMA are reduced when engaging in motor activity relative to when passively viewing others’ pain^52^. Our findings further link representations of empathic pain in SMA to increased altruistic motivation and the perception of others’ pain.

Our findings are also consistent with previous work investigating the relationship between altruism and empathic responses in AI. Tusche and colleagues found that charitable donations can be predicted from activity patterns in AI in individuals whose decisions are most influenced by feelings of empathy^48^. Furthermore, AI activation while observing others’ social exclusion correlates with trait empathy and efforts to console the excluded individual^53^. Our prior work has revealed extraordinary altruists’ heightened neural sensitivity to the distress of strangers in various affective regions including the AI, amygdala and midbrain^3–5^. Through the use of MVCC, the current findings extend this work and answer the recently raised question^54^ of whether altruists’ sensitivity to others’ distress in AI reflects responses that are representationally similar to their own experience of distress.

Some limitations should be considered in interpreting these findings. The paradigm required the empathy prompt condition to follow the spontaneous empathy condition, as prior prompting would preclude measurement of spontaneous empathic responses. The possibility of order effects should therefore be considered. However, we found no evidence of empathic habituation in reported perceptions of the partner’s pain. We also designed the experiment so that subjects completed the empathy conditions prior to their first-hand experience of pain to reduce potential carryover effects from repeated exposure to the painful stimulus, which could have volatile effects on empathic responses. It is of course possible that subjects varied in their interpretation and response during the empathic epochs; for example, during the fearful anticipation epoch, some subjects may have anticipated their own future empathic responding for the partner’s pain. Altruists were also calibrated to a higher pain stimulus level in order to reach a rating of “slightly intense” pain. This difference was unexpected, but notably, neuroimaging results were unaffected by inclusion of PSI level as a covariate. Potential group differences in pain sensitivity could be associated with altruists’ history of undergoing surgery; however, these results should be interpreted with caution as our pain administration technique was not optimized to evaluate variation in pain sensitivity. It is also possible that other factors unrelated to altruism (e.g. geographical location) distinguish altruistic donors and controls and should be considered when assessing the generalizability of our findings.

An additional consideration is that multivariate fMRI analyses are subject to ongoing methodological and theoretical discussions^23^. While we bidirectionally trained and tested classifiers for each context comparison and averaged the result, investigation into how directional training might affect classifier performance in empathy paradigms may be of interest in future research. It should be noted that a particular strength of this study is its focus on evaluating neural responses in a sample of real-world costly altruists. By evaluating altruistic kidney donors—a population associated with elevated prosocial tendencies across a variety of situations, including frequent blood donation and volunteering^55^—our findings capture an ecologically valid and stringently defined form of altruism, while simultaneously minimizing confounds associated with laboratory-induced and self-reported altruism, including social desirability and self-presentation biases^56^.

In conclusion, this study provides important new information on the neural correlates of altruism. By revealing significant differences in altruists’ responses to the pain and fearful anticipation of strangers, our results suggest that real-world, costly altruistic behaviour is associated with both enhanced tendencies and proclivities for affective resonance with strangers’ distress, in particular, during spontaneous responses to others’ fearful anticipation in left AI, spontaneous responses to others’ pain in right AI, dACC/aMCC and SMA, and prompted responses to others’ fearful anticipation in dACC/aMCC. These findings also reinforce the importance of empathy not only for sensory states like pain but for anticipatory affective states like fear, and the importance of differentiating between an individual’s proclivity versus ability to empathize with the pain and distress of others.

## Methods

To address our first goal—evaluating the role of spontaneous affective resonance in altruism—we used MVCC to compare patterns of responses during experienced and observed fearful anticipation and, separately, experienced and observed pain. These analyses were conducted using data initially analyzed to assess univariate BOLD responses and functional connectivity during the first-hand experience of distress and the spontaneous observation of others’ distress^5^. To address our second goal—evaluating prompted empathic responses in altruism—we used MVCC to compare patterns of brain responses collected during the first-hand experience of distress and the observation of others’ distress following an empathy prompt. Data collected during this empathy prompt condition have never been previously analyzed or published.

### Subjects

Fifty-seven subjects took part in this study, including 29 altruistic kidney donors and 28 matched controls. Altruistic donors were recruited from across North America via transplant organizations and online advertisements and all donations were verified through independent sources (e.g. transplant center records, news articles). Controls were recruited from the Washington, DC area using flyers, online advertisements, and the ResearchMatch database. Interested individuals completed an online screening measure that inquired about variables relevant to eligibility including demographics, altruistic donation, and MRI contraindications.

Researchers coordinated travel and lodging for altruists who lived more than a two-hour drive from Georgetown University. On-site testing included functional magnetic resonance imaging (fMRI) scanning as well as assessments of cognitive ability using the Kaufman Brief Intelligence Test – Second Edition^57^, demographic information, psychological history, medication use, and handedness. All study procedures were carried out in accordance with a protocol approved by the Institutional Review Board at Georgetown University in Washington, DC, and participants provided written informed consent. Exclusion criteria included MRI contraindication, pain disorders, hearing difficulties, IQ < 80, use of psychotropic medication within the past two weeks, history of head injury or neurological illness, or clinically significant psychopathology as indexed by scores above clinical cutoffs for Global Severity, Positive Symptom Distress, or Positive Symptom Total on the Symptom Checklist–90^58^ (excluding elevated total scores due to the interpersonal sensitivity or hostility subscales). For controls, additional exclusion criteria included having volunteered to be a living organ donor or indicating interest in learning about becoming a living organ donor in response to a screening question.

Three altruists and one control subject were excluded for excessive motion during the MRI scan (>15% TRs with >0.5 mm head-movement). One additional altruist was excluded for not completing the full scanning session, resulting in a final sample of 25 altruistic kidney donors and 27 controls. Groups did not differ in age, sex, race, IQ, or level of education (Table 1). Our altruistic kidney donor sample was generally representative of the national non-directed kidney donor population in terms of sex and race (national sample through 2016: 56% female, 93% white; Organ Procurement and Transplantation Network Data as of July, 2018). All subjects received compensation for their participation.

**Table 1:** Subject demographics.

	Altruists	Controls	*p-value* ^[a]^	BF_10_^[b]^
N	25	27		
Age *M* (*SD*)	41.9 (9.9)	38. 9 (8.0)	0.23	0.51
IQ *M* (*SD*)	108.3 (12.4)	111.2 (11.3)	0.38	0.39
Male/Female (% Male)	9/16 (36.0%)	13/14 (48.1%)	0.41	0.48
White/Other Race (% White)	23/2 (92.0%)	22/5 (81.5%)	0.42	0.40
Education ≥4-Year Degree (%)	17 (68.0%)	24 (88.9%)	0.09	1.39

Note: ^[a]^*p-*value (two-tailed) is based on Fisher’s exact test for categorical variables and two-sample t-tests for continuous variables. ^[b]^Bayes factors (BF_10_) indicate the probability of the data given the alternative hypothesis relative to the null hypothesis (i.e. values larger than 1 support the alternative).

To evaluate whether our groups differed in terms of other ecologically valid forms of altruism, we inquired about blood donation and charitable volunteering in the online screening survey. A Fisher’s Exact Test revealed that, in our final sample, more altruists reported previously donating blood (Altruists: 92%; Controls: 63%; p = 0.020). And of the subjects who reported donating blood, altruists reported donating on more occasions (Altruists: Mdn = “10–15” times, range = “1–5” to “more than 20”, n = 23; Controls: Mdn = “1–5” times, range = “1–5” to “more than 20”, n = 17; *U* = 313.5, p < 0.001). At a trend level, altruists reported a greater frequency of charitable volunteering (Altruists: Mdn = “2–3 times a month”, range = “Never” to “Daily”; Controls: Mdn = “Less than once a month”, range = “Never” to “2–3 times a week”; *U* = 438.5, p = 0.058).

### Functional neuroimaging task

Prior to scanning, pressure-pain stimulation was calibrated for each participant, which was applied to their right thumbnail and administered by a computerized device that maintained constant pressure for 6 seconds. Pneumatic pressure per square inch (PSI) was titrated to a pain level that was reported to be “slightly intense” for each subject prior to the task (rated 13.5 on a 21-point Gracely Box Scale^59^). Then, upon entering the console room outside of the scanner, participants were briefly introduced to a female stranger described as their study partner, who was actually a trained confederate and who did not converse with participants before they were escorted into the magnet. At the time of introduction, the study partner was visibly connected to the pain stimulus device.

The task consisted of three runs, each lasting 12 m 18 s and containing 15 aversive trials and 15 non-aversive trials. The first run contained pain events only for the study partner, the second run also contained pain events only for the partner and additionally included a verbal empathy prompt prime, which was provided to the participant through in-scanner headphones. The third run contained pain events only for the participant. In all runs, pain stimulus onset was cued by a 1 s neutral tone, which followed an anticipation period of 6, 9, 12, or 15 s that was cued by a distinct 1 s neutral tone. Safety tones were used to create non-aversive control trials that otherwise matched the variable-length anticipation and fixed-length pain events. Inter-trial intervals lasted 3, 6, 9 or 12 s. Subjects learned the significance for each of the four tones prior to the task. Auditory and pressure stimuli presentation was controlled by E-prime 2.0 (Psychology Software Tools, Pittsburgh, PA). The pain stimulus was omitted on one third of aversive trials so that pain cues were probabilistic, which has been shown to optimally promote fearful anticipation^60^.

During scanning, participants viewed live video feeds of the hand of their study partner during the first and second runs and their own hand during the third run. Imagery was visually matched across runs with both the participant’s hand and the study partner’s hand viewed from similar angles against a black cloth background. The PSI level for the study partner was held constant across participants (15 PSI), which was within the range of levels selected by participants. The study partner heard the same tones as the subject and was fully aware of the task paradigm.

### Subjective ratings

Immediately following each run, participants reported their perception of pain using a 7-point scale (1: No pain to 7: Extreme pain). This scale separately indexed the perceived pain of the study partner after runs 1 and 2 and experienced pain after run 3.

After completion of the scan, participants retrospectively rated how unpleasant the aversive trials were, and how fearful/anxious they felt during the anticipation period using 7-point scales (1: Not at all to 7: Extremely). Participants also answered the same questions regarding the experience of their study partner overall, which collapsed across runs 1 and 2. One control subject did not complete the post-scan questionnaire resulting in a subsample of 25 altruists and 26 controls for this questionnaire.

### fMRI data acquisition

Anatomical and functional brain images were acquired with a 3T Siemens TIM Trio scanner and a 12-channel phased-array head coil. T1-weighted MP-RAGE anatomical images were obtained for each subject (176 1 mm axial slices; field of view, 250 mm^2^; repetition time, 1,900 ms; echo time, 2,520 ms; 256 × 256 matrix; 1 × 1 × 1 mm voxels). T2*-weighted functional images were collected using an echo-planar imaging sequence (46 3 mm transversal slices; repetition time, 2,500 ms; echo time, 30 ms; field of view, 192 mm^2^; 64 × 64 matrix; 3 × 3 × 3 mm voxels).

### fMRI data analysis

Preprocessing of functional images was completed in AFNI^61^. The first 4 volumes of each run were removed and remaining images were despiked, slice-time corrected, aligned to the subject anatomical grid and motion-corrected. Motion artifacts were modeled using six rigid-body motion parameters and were included in the regression model for each subject. Low frequency signal drifts (>100 s) were removed and AFNI’s 3dLSS least-squares-sum regression was used to obtain parameter estimates for each event^62^. Each run contained 15 fearful anticipation events, 15 no-fearful anticipation events, and, due to the probabilistic nature of the pain stimulus, 10 pain events and 20 no-pain events.

Analyses were performed on unsmoothed parameter estimate images in subject anatomical space using The Decoding Toolbox^63^. All analyses used a searchlight with a radius of 3 voxels (9 mm) constrained within a gray matter mask, which was obtained by warping the gray matter mask of the ICBM 2009c nonlinear symmetric template^64^ into each subjects’ anatomical space. Nonlinear warp parameters were obtained using 3dQwarp (Cox & Glen, 2013) and the ICBM 2009c nonlinear symmetric template.

A linear SVM classifier was trained on neural response patterns during one context and tested on response patterns during a different context (i.e. trained on *experienced* pain versus no-pain then tested on *observed* pain versus no-pain). We performed each cross-classification analysis twice, bidirectionally training and testing each context and averaging the result, consistent with prior approaches^17,26,27^. To evaluate classifier performance, we calculated AUC, a sensitive metric that reduces potential bias due to unbalanced classes^65^. For each cross-classification analysis, the center voxel of each searchlight was assigned the AUC value from that spherical region of interest and chance level was subtracted. Subjects’ AUC maps were subsequently warped to standard space and spatially smoothed with a 6 mm full-width half maximum Gaussian filter.

For the purpose of conducting small volume correction (SVC) in *a priori* regions of interest, masks were used to define AI and dACC/aMCC using modifications of the automated anatomical labeling atlas^66^. Right and left AI masks included insular labeled regions, and were constrained to be anterior to y = 0^67^. For the dACC/aMCC mask, both the anterior cingulate and the middle cingulate cortex labeled regions were included, and were also constrained to be anterior to y = 0 based on Neurosynth reverse inference maps for “fear” and “pain”^68,69^.

For visualization purposes, SVM pattern weights were obtained using the SVM_pattern output function when assessing classification in the whole brain gray matter mask. This function multiplies raw SVM weights by the covariance of the data^70,71^.

### Statistical analyses

All reported measurements were taken from a single sample of subjects. Sample size was determined using fMRIPower based on pilot data from a previous study of altruistic kidney donors^3^. A between-subject design probed cross-classification differences between the final sample of altruistic kidney donors and controls using two-tailed, two-sample t-tests in the whole brain and within three *a priori* regions of interest using SVC (right AI, left AI and dACC/aMCC). All statistical neuroimaging analyses applied a permutation approach to determine cluster-size thresholding via the -Clustsim flag in AFNI’s 3dttest++, which randomizes and permutes input datasets using 10,000 Monte Carlo simulations. This approach was developed to reduce the false positive rate in response to Eklund *et al*.^72,73^. Cluster significance was determined using an underlying voxel height threshold of p < 0.001 and a cluster forming threshold to control the false positive rate at p < 0.05. Within-group analyses used two-tailed, paired sample t-tests to test the classifiers’ ability to discriminate between aversive and non-aversive events across experienced and observed contexts different than chance. Analyses of demographic and self-report data used two-tailed, two-sample t-tests to examine group differences on continuous variables and two-tailed Fisher’s exact tests to examine group differences on categorical variables. Bayesian analyses of demographic variables were conducted in JASP Version 0.9.2 using default priors (JASP Team, 2018). To investigate changes in observed pain ratings before and after the empathy prompt we used a 2 × 2 mixed ANOVA and report effect size as partial eta squared. All other effect sizes were calculated using Cohen’s d and report the corresponding 95% CI.

## Supplementary information

Supplementary Materials

## Data Availability

Non-identifying demographic data, self-report responses, statistical maps and analysis scripts are publicly available on the Open Science Framework (osf.io/ubzfk). Age of participants has been withheld to protect identity.
