# Supplementary material for: Increased similarity of neural responses to experienced and empathic distress in costly altruism
Source: Sci Rep. 2019 Jul 24;9:10774. doi: 10.1038/s41598-019-47196-3 (PMC6656917; doi:10.1038/s41598-019-47196-3)
Supplement: Supplementary file 1 — Supplementary Materials [file 41598_2019_47196_MOESM1_ESM.pdf]

Supplementary Information

Increased similarity of neural responses to experienced and empathic distress in costly altruism

Katherine O'Connell, Kristin M. Brethel-Haurwitz, Shawn A. Rhoads, Elise M. Cardinale, Kruti M. Vekaria, Emily L. Robertson, Brian Walitt, John W. VanMeter, & Abigail A. Marsh

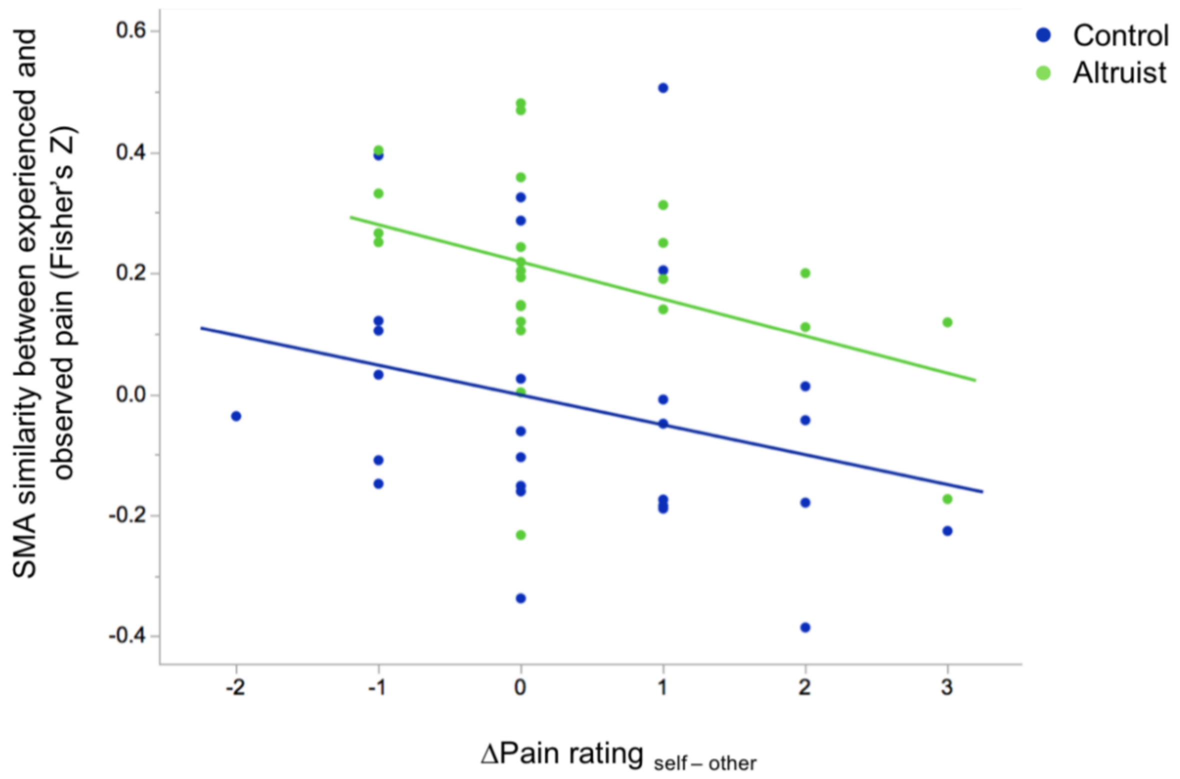

Supplementary Fig. 1: Subjects who reported their own pain to be more intense than their partners' pain exhibited reduced similarity between SMA responses to experienced and observed pain. (See Supplemental Text)

Supplementary Table 1. Cross-classification between experienced and observed fearful anticipation. (Related to Figure 2a)

k	X	Y	Z	Peak Z	AUC	Hemi	Peak Region
Altruists							
127	8	53	23	4.87	0.57	R	Sup. frontal gyrus (BA9)
94	2	-50	44	4.81	0.57	R	Precuneus
92	41	26	-2	5.15	0.58	R	AI/IFG (BA45)
89	-26	-98	17	4.60	0.59	L	Mid. occipital gyrus
41	-38	17	5	4.82	0.57	L	AI/IFG (BA44)
41	38	-62	56	4.62	0.57	R	Sup. parietal lobule (BA7)
39	-53	-56	26	4.89	0.57	L	Angular gyrus (BA39)
35	-2	-14	44	4.06	0.57	L	Post. cingulate
34	-20	-83	-23	4.70	0.58	L	Cerebellum
29	-59	-8	-14	4.34	0.57	L	Mid. temporal gyrus
25	5	-86	5	4.64	0.57	R	Cuneus
Controls							
228	47	23	5	5.30	0.58	R	AI/IFG (BA45)
92	62	-50	23	4.59	0.57	R	Angular gyrus (BA39)
84	5	14	56	5.07	0.57	R	Sup. frontal gyrus (BA8)
54	65	-44	11	4.29	0.56	R	Sup. temporal gyrus (BA22)
43	44	35	38	4.64	0.57	R	Mid. frontal gyrus (BA9)
38	8	-59	50	4.21	0.57	R	Precuneus
30	65	-20	20	5.24	0.56	R	Supramarginal gyrus (BA40)
Altruists > Controls							
3	-32	14	8	4.22[a]		L	AI

Note: Results are reported in MNI space using a threshold of p<.05 corrected for multiple comparisons at a voxel height threshold of p<.001 in the whole brain or [a] using small-volume correction in group comparisons. BA, Brodmann area; k, cluster size in 3 mm^3 voxels; L, Left; R, Right; AUC, mean area under the curve within the cluster (chance = 0.5); IFG, inferior frontal gyrus; AI, anterior insula.

Supplementary Table 2. Cross-classification between experienced and observed pain. (Related to Figure 4a)

k	X	Y	Z	Peak Z	AUC	Hemi	Peak Region
Altruists							
5278	62	-20	38	7.21	0.64	R	Supramarginal gyrus (BA40)
4322	-53	-74	2	6.82	0.63	L	Mid. occipital gyrus
334	5	14	65	5.08	0.60	R	Sup. frontal gyrus (BA6)
254	-5	-50	23	5.06	0.59	L	Post. cingulate
233	-2	-26	47	5.23	0.60	L	Post. cingulate
189	-44	47	20	5.20	0.59	L	Mid. frontal gyrus (BA10)
138	2	65	20	5.16	0.58	R	Sup. frontal gyrus (BA10)
130	-23	-74	-56	4.78	0.61	L	Cerebellum
113	5	-65	65	5.10	0.60	R	Precuneus
54	14	-74	-53	4.52	0.60	R	Cerebellum
53	11	14	-8	4.89	0.58	R	Nucleus accumbens
Controls							
5789	62	-29	29	7.35	0.62	R	Supramarginal gyrus (BA40)
1341	-62	-26	26	6.73	0.63	L	Supramarginal gyrus (BA40)
134	14	-35	2	5.75	0.58	R	Thalamus
118	-62	8	23	5.18	0.59	L	Precentral gyrus (BA4)
111	-17	-71	-47	5.49	0.59	L	Cerebellum
109	-20	-86	-17	4.67	0.59	L	Lingual gyrus
64	14	-80	-20	5.46	0.58	R	Cerebellum
63	56	35	8	4.49	0.58	R	IFG (BA45)
41	-35	-5	62	4.48	0.59	L	Precentral gyrus (BA4)
Altruists > Controls							
84	-5	-5	68	4.92		L	Sup. frontal gyrus (SMA; BA6)
6	5	47	23	3.99[a]		R	Ant. cingulate
4	35	14	2	3.89[a]		R	AI
2	35	20	-11	3.41[a]		R	AI

Note: Results are reported in MNI space using a threshold of $p<.05$ corrected for multiple comparisons at a voxel height threshold of $p<.001$ in the whole brain or [a] using small-volume correction in group comparisons. BA, Brodmann area; k, cluster size in 3 mm^3 voxels; L, Left; R, Right AUC, mean area under the curve within the cluster (chance = 0.5); IFG, inferior frontal gyrus; AI, anterior insula.

Supplementary Table 3. Cross-classification between experienced and observed fearful anticipation after the empathy prompt. (Related to Figure 2b)

k	X	Y	Z	Peak Z	AUC	Hemi	Peak Region
Altruists							
315	8	-71	35	4.92	0.58	R	Precuneus
141	2	-80	-5	5.30	0.59	R	Lingual gyrus
128	-35	-92	-14	5.33	0.58	L	Inf. occipital gyrus
116	38	29	-5	4.89	0.58	R	AI/IFG (BA47)
109	5	38	29	4.88	0.57	R	Sup. frontal gyrus (BA8/9)
93	-50	-59	26	4.95	0.57	L	Angular gyrus (BA39)
82	-62	-38	35	4.43	0.57	L	Supramarginal gyrus (BA40)
77	59	-41	26	5.12	0.58	R	Supramarginal gyrus (BA40)
66	-50	-5	53	5.04	0.58	L	Precentral gyrus (BA4)
62	47	8	47	4.91	0.58	R	Precentral gyrus (BA4)
61	53	-71	17	4.73	0.57	R	Mid. occipital gyrus
51	35	-80	20	4.94	0.58	R	Mid. occipital gyrus
44	-17	-77	-23	4.39	0.58	L	Cerebellum
33	44	-44	47	4.59	0.56	R	Supramarginal gyrus (BA40)
32	29	-98	11	4.03	0.58	R	Cuneus
32	-29	-53	50	4.39	0.57	L	Sup. parietal lobule (BA7)
28	29	62	-11	4.08	0.57	R	Sup. frontal gyrus (BA10)
28	2	56	-2	4.13	0.58	R	Sup. frontal gyrus (BA10)
26	-38	62	2	4.03	0.58	L	Sup. frontal gyrus (BA10)
Controls							
78	50	20	8	5.05	0.57	R	AI/IFG (BA47)
67	71	-29	-8	4.58	0.57	R	Mid. temporal gyrus (BA21)
26	44	2	56	4.10	0.57	R	Precentral gyrus (BA4)
Altruists > Controls							
43	2	-80	-5	4.76		R	Lingual gyrus
5	-11	23	38	3.73[a]		L	Ant. cingulate

Note: Results are reported in MNI space using a threshold of $p<.05$ corrected for multiple comparisons at a voxel height threshold of $p<.001$ in the whole brain or [a] using small-volume correction in group comparisons. BA, Brodmann area; k, cluster size in 3 mm^3 voxels; L, Left; R, Right; AUC, mean area under the curve within the cluster (chance = 0.5); IFG, inferior frontal gyrus; AI, anterior insula.

Supplementary Table 4. Cross-classification between experienced and observed pain after the empathy prompt. (Related to Figure 4b)

k	X	Y	Z	Peak Z	AUC	Hemi	Peak Region
Altruists							
5796	62	-32	38	6.87	0.62	R	Supramarginal gyrus (BA40)
2495	-47	-80	-2	6.51	0.62	L	Mid. occipital gyrus
192	2	26	62	4.67	0.59	R	Sup. frontal gyrus (BA8)
140	5	62	26	4.77	0.59	R	Sup. frontal gyrus (BA10)
128	-2	-32	38	4.55	0.58	L	Post. cingulate
99	-56	14	17	4.87	0.60	L	IFG (BA44)
88	-11	2	14	5.01	0.58	L	Caudate
83	-2	23	-17	4.91	0.59	L	Subgen. cingulate
48	-47	14	-2	4.51	0.60	L	AI/IFG (BA44)
35	-53	2	50	4.08	0.59	L	Precentral gyrus (BA4)
Controls							
3101	53	-62	11	6.65	0.63	R	Mid. occipital gyrus
1476	-50	-71	2	6.10	0.63	L	Mid. occipital gyrus
410	56	11	17	5.64	0.60	R	Precentral gyrus (BA4)
175	-5	-53	20	4.69	0.59	L	Post. cingulate
89	44	2	53	5.04	0.60	R	Precentral gyrus (BA4)
55	-17	-71	-50	4.79	0.59	L	Cerebellum
51	-5	-50	62	4.34	0.59	L	Precuneus
35	-2	-41	47	4.57	0.58	L	Precuneus
Altruists > Controls							
No significant differences							

Note: Results are reported in MNI space using a threshold of $p<.05$ corrected for multiple comparisons at a voxel height threshold of $p<.001$ in the whole brain. BA, Brodmann area; k, cluster size in 3 mm^3 voxels; L, Left; R, Right; AUC, mean area under the curve within the cluster (chance = 0.5); IFG, inferior frontal gyrus; AI, anterior insula.

Supplementary Table 5. Correlation coefficients for Δpain rating $_{self-other}$ and degree of similarity between experienced and observed pain responses.

	SMA	Right AI	Left AI	dACC/aMCC
Across Groups (n=52)	**-.288*** (.039)	**.088** (.533)	**-.163** (.248)	**.000** (.999)
Altruists (n=25)	**-.456*** (.022)	**.256** (.216)	**.029** (.891)	**.328** (.109)
Controls (n=27)	**-.370**† (.057)	**-.057** (.778)	**-.361**† (.064)	**-.358**† (.067)

Note: Table reports Spearman's rho followed by the uncorrected p-value in parentheses; $^{\dagger}p<.10_{uncorrected}$; * $p<.05_{uncorrected}$; SMA, supplementary motor area; AI, anterior insula; dACC/aMCC, dorsal anterior cingulate cortex/anterior midcingulate cortex.

Supplemental Text

Relationship between self-other neural similarity to pain and subjective pain ratings

We evaluated whether the similarity of subjective perceptions of experienced and observed pain correspond to the similarity of brain response patterns during experienced and observed pain in key regions of interest, including AI, dACC/aMCC, and SMA.

Mean voxel-wise responses to experienced pain and observed pain were calculated for each subject by averaging unsmoothed parameter estimates in subject anatomical space, which were then subtracted by mean response to their respective non-aversive control events. Within regions of interest, values for each voxel were extracted using 3dmaskdump. Vectorized voxel response values for experienced and observed pain>no-pain were then passed through a hyperbolic tangent function before being correlated to obtain a single Pearson r value for each subject and each region representing response similarity between experienced and observed pain. Obtained r values were Fisher-Z transformed and submitted to a two-tailed Spearman correlation test with pain perception difference scores (Δpain rating $_{self - other}$). The SMA region of interest was defined using the 84-voxel cluster resulting from the group comparison analysis for experienced and observed pain cross-classification.

No significant correlations with Δpain rating $_{self - other}$ were found for AI or dACC/aMCC. In SMA, a non-parametric correlation test across all subjects revealed a relationship between the similarity of experienced and observed pain representations in SMA and Δpain rating $_{self - other}$ (Supplementary Fig. 1). Subjects who reported experiencing more intense pain than their partner exhibited *reduced* similarity between experienced and observed pain responses in SMA (rho=-.288, p_{uncorr}=.039). This relationship was also observed when considering each group separately, although at a trend level in controls (Altruists: rho=-.456, p_{uncorr}=.022; Controls: rho=-.370, p_{uncorr}=.057). All correlations are reported in Supplementary Table 5.
